# Rapidly destructive osteoarthritis of the spine: lessons learned from the first reported case

**DOI:** 10.1186/s12891-022-05686-y

**Published:** 2022-08-01

**Authors:** Alice Baroncini, Pedro Berjano, Filippo Migliorini, Claudio Lamartina, Daniele Vanni, Stefano Boriani

**Affiliations:** 1 IRCCS Istituto Ortopedico Galeazzi, Milano, Italy; 2 Department of Orthopaedics and Trauma Surgery, RWTH Aachen University Clinic, Aachen, Germany

**Keywords:** Rapidly Destructive Osteoarthritis, Rapidly Progressive Osteoarthritis, Spine, Spinal RDOA, Lumbar Interbody Cage, 4-Rod Construct

## Abstract

**Background:**

Rapidly Destructive Osteoarthritis (RDOA) has been described for the hip and shoulder joints and is characterized by a quickly developing bone edema followed by extensive remodeling and joint destruction. Confronted with a similarly evolving case of endplate edema and destruction of the disk space, we offer the first described case of spinal RDOA and illustrate the challenges it presented, along with the strategies we put in place to overcome them.

**Case presentation:**

We present a case of spinal RDOA that, also due to the delay in the diagnoses, underwent multiple revisions for implant failure with consequent coronal and sagittal imbalance. A 37-years-old, otherwise healthy female presented with atraumatic low back pain: after initial conservative treatment, subsequent imaging showed rapidly progressive endplate erosion and a scoliotic deformity. After surgical treatment, the patient underwent numerous revisions for pseudoarthrosis, coronal and sagittal imbalance and junctional failure despite initially showing a correct alignement after each surgery. As a mechanic overload from insufficient correction of the alignement of the spine was ruled out, we believe that the multiple complications were caused by an impairment in the bone structure and thus, reviewing old imaging, diagnosed the patient with spinal RDOA. In case of spinal RDOA, particular care should be placed in the choice of extent and type of instrumentation in order to prevent re-intervention.

**Conclusion:**

Spinal RDOA is characterized by a quickly developing edema of the vertebral endplates followed by a destruction of the disk space within months from the first diagnosis. The disease progresses in the involved segment and to the adjacent disks despite surgical therapy. The surgical planning should take the impaired bone structure account and the use of large interbody cages or 4-rod constructs should be considered to obtain a stable construct.

## Background

Rapidly destructive osteoarthritis (RDOA, a.k.a. rapidly progressive osteoarthritis, RPOA) is a rare clinical entity that has so far been described only for the hip and shoulder joints [1]. RDOA presents with a quick onset and evolution that is clearly shown in radiographic imaging, which is the only diagnostic tool of this disease [1], along with the exclusion of other causes of rapidly evolving joint disease such as sepsis, rheumatoid arthritis, crystalline arthropathy or osteonecrosis [1]. RDOA is a rare entity, with only 181 cases reported so far for the hip joint [2].

As mentioned, RDOA has so far been observed only in the hip and shoulder joint. Here we present the first described case of spinal RDOA. The patient, an otherwise healthy, young female, presented with low back pain and rapidly evolving degenerative changing in the radiographic imaging. Despite a surgical management that correctly restored the sagittal and coronal alignement, the patient presented multiple failures and underwent multiple revisions. The peculiar clinical course lead to a retrospective review of the case and, after comparison with the available data on hip and shoulder, to the diagnosis of spinal RDOA. The recognition of this clinical setting and of the challenges it presents to the surgeon are of paramount importance for a careful therapeutic planning and a successful treatment.

### Case presentation

The case is presented according to the Case Report (CARE) guidelines [3].

**Fig. 1:**
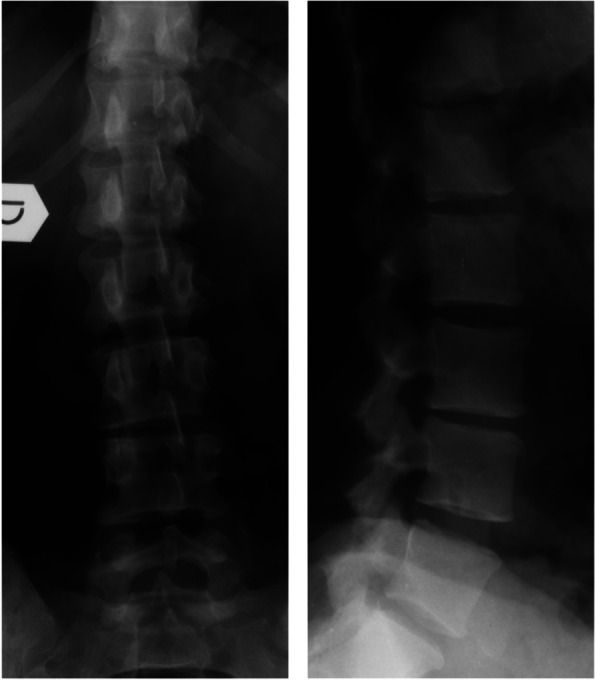
X-rays of the patient at the first consult in June 2002

**Fig. 2:**
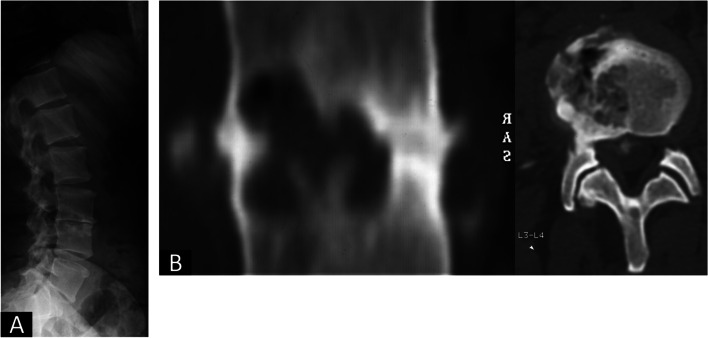
**A **and** B**: The imaging obtained in September 2003 showed a rapidly evolving degeneration of the L3/4 disk space (**A**) with an erosion of the L3 and L4 endplates in the CT scan (**B**)

An otherwise healthy, 37 year old female patient consulted our outpatient clinic in June 2002 for a newly arose, atraumatic low back pain. The x-rays showed an early-stage degenerative scoliotic curve (Fig. 1) and conservative treatment with painkillers and physiotherapy was begun. The patient sought consultation again in September 2003 due to an increase of the low back pain associated with left sciatic pain without neurological deficits: the x-rays and CT scan showed a marked progression of the deformity with endplate erosion (Fig. 2). Further diagnostics through imaging (MRI, bone scintigraphy) and blood tests could rule out a septic, malignant or rheumatic process, which could have offered an explanation for the rapid evolution of the deformity and for the endplate erosion. In particular, leucocyte count and C-reactive protein were within normal values, as well as the rheumatoid factor, the anti-cyclic citrullinated peptide and the erithrocyte sedimentation rate.

**Fig. 3:**
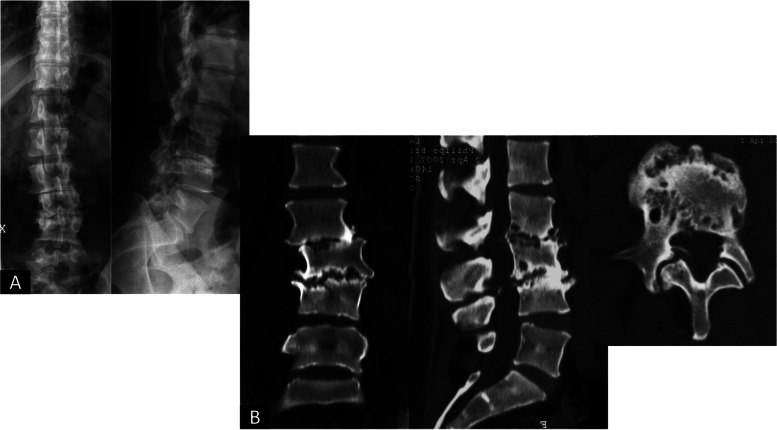
**A **and** B**: Evolution of the deformity in the x-rays and increased endplate erosion (**A**), which involved L2 as well as shown in the CT scan (**B**-April 2007)

**Fig. 4:**
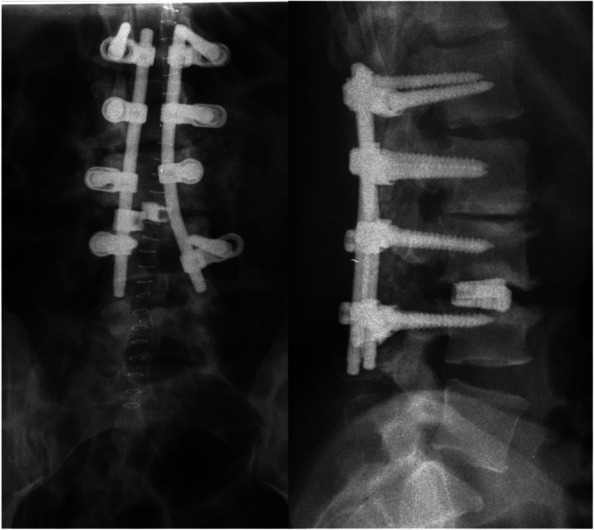
The postoperative x-rays showed a correct implant positioning after L1-L4 fusion with L3/4 PLIF (May 2007)

The symptoms and the endplate erosion further worsened in April 2007 (Fig. 3). Due to severe functional impairment, the patient was scheduled for surgery with the diagnosis of Aebi I (de novo) scoliosis [4] secondary to rapidly progressive disk degeneration – however, a precise diagnosis for this fast-evolving process could not be pinpointed. In May 2007 a L1-L4 posterior fusion with L3/4 PLIF was performed (Fig. 4). A microbiological analysis of the intervertebral disc confirmed the absence of infection. One year after surgery the patient reported a reduction in pain levels and could go back to moderate physical activity.

**Fig. 5:**
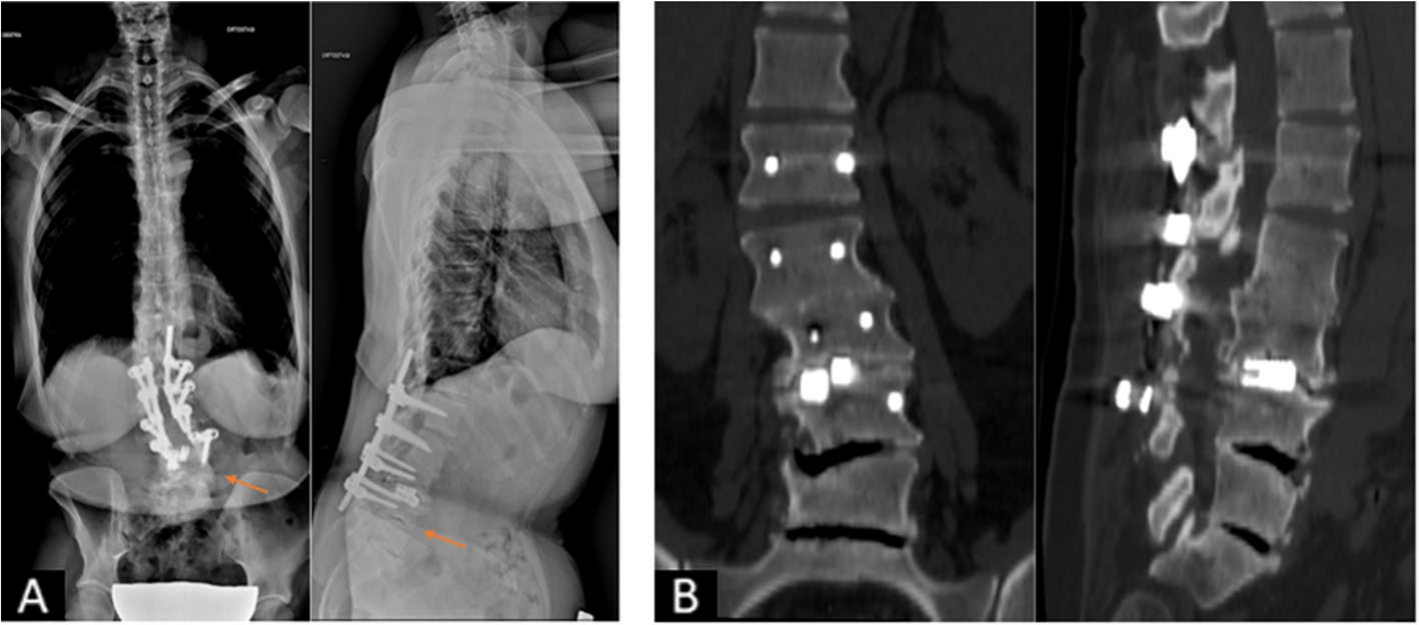
**A **and** B**: The x-rays (**A**) and CT scan (**B**) obtained in December 2012 showed a DJK an an erosion of the L5 endplate. Despite an anterior bony fusion from L2 to L4, the PLIF cage was clearly subsided, possibly due to the impaired bone structure in the endplates of L3 and L4

**Fig. 6:**
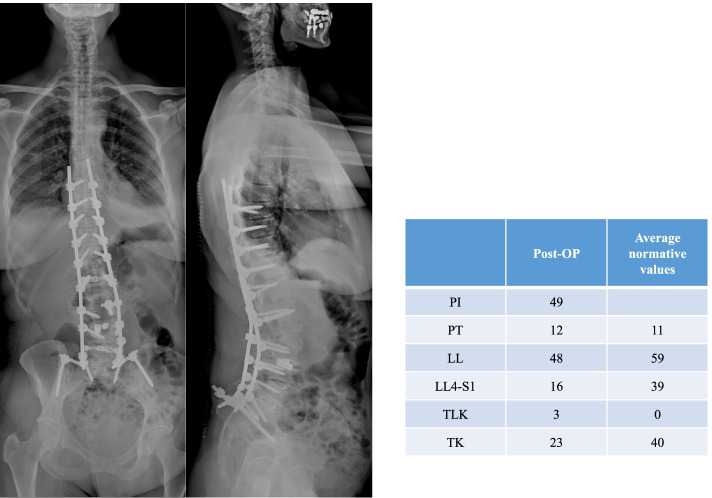
Postoperative x-ray following revision surgery in December 2012. A T8-pelvis instrumentation with a pedicle subtraction osteotomy in L2 and a TLIF L4/5 and L5/S1 was performed. The revision allowed correction of the DJK and restoration of the sagittal profile to values approaching the normative ones (*PI* Pelvic incidence, *PT* Pelvic tilt, *LL* Lumbar lordosis L1-S1, *LLL* Low lumbar lordosis L4-S1, *TLK* Thoracolumbar kyphosis T10-L2, *TK* Thoracic kyphosis T1-T12; all measurements are in degrees)

In December 2012 the patient consulted again for increasing back pain and sagittal imbalance, and a distal junction kyphosis (DJK) was diagnosed (Fig. 5). A surgical revision was conducted in the same month and allowed to restore a physiological sagittal alignment (Fig. 6).

**Fig. 7:**
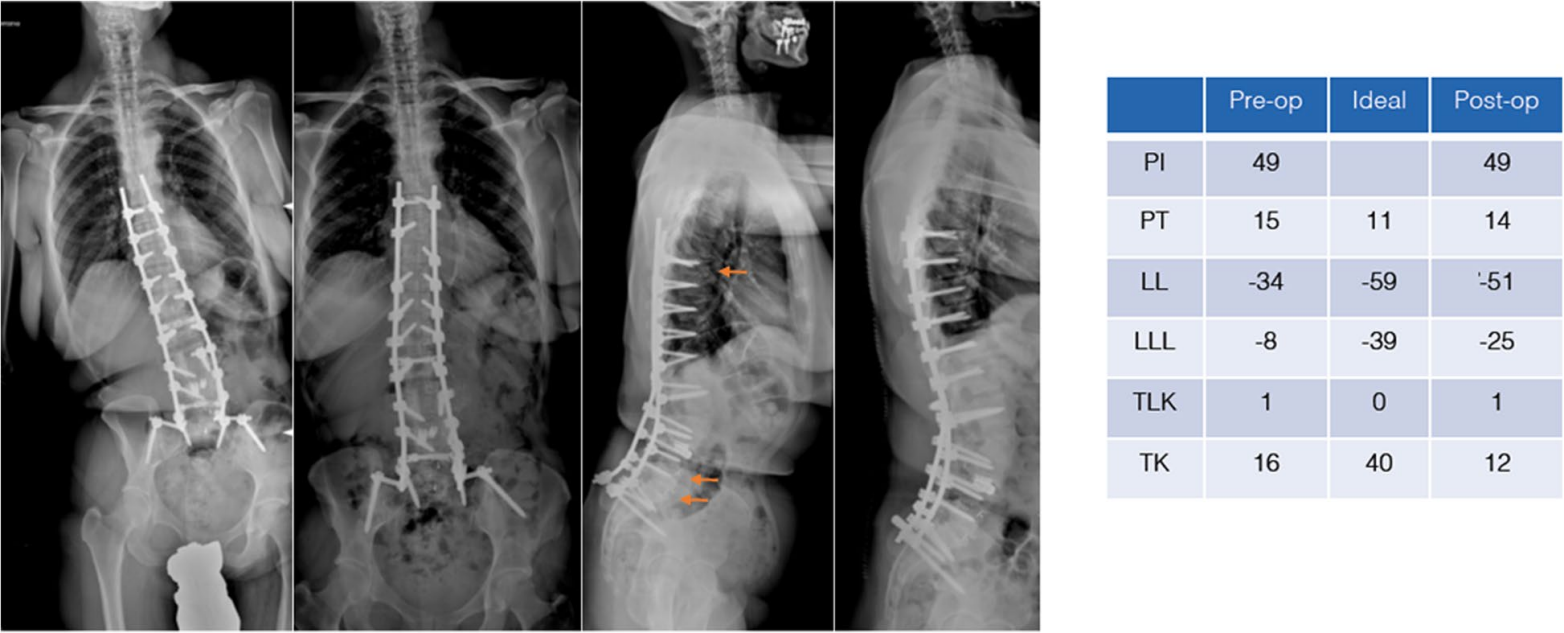
Pre- and postoperative x-ray following revision surgery in March 2013. Before surgery, a T8 fracture and a subsidance of the TLIF cages had been diagnosed (arrows). A T7-pelvis revision with implant renewal was performed. Again, the postoperative sagittal parameters were comparable with normative values with the exception of TK

Shortly after surgery (March 2013), however, the patient presented again with increasing pain and coronal and sagittal imbalance. The x-rays and CT scan showed a subsidence of both TLIF cages, along with a T8 fracture. For this reason, a posterior revision with a T7-pelvis instrumentation was performed. After surgical revision a satisfactory sagittal balance was obtained again (Fig. 7).

Unfortunately, in October 2019 the patient consulted again for a relapse of the lumbar pain and sagittal imbalance. In the meantime, an open reduction and internal fixation of the left proximal femur had been performed due to accidental trauma. An osteoporosis evaluation with bone densitometry and blood tests (creatinin, vitamin D, phosphate, TSH and calcium) had been performed after the femur fracture and could rule out an osteoporotic process. Due to the successive and multiple implant failure, the case was reviewed to search for a possible explanation for this particular clinical course. The patient still did not present signs of rheumatic, septic or tumorous illness and the blood tests did not show any alteration. The available imaging was compared with the literature on hip and shoulder joints, leading to test the hypothesis of spinal RDOA, which is currently based on radiographic findings. RDOA first presents with bone marrow edema and subchondral fractures, which are evident in Fig. 2 B. This phase is followed by extensive bone remodelling and osteophyte formation as shown in Fig. 3. We believe that these observation are consistent with the diagnosis of spinal RDOA. This diagnosis offers an explanation for the quick evolution of the symptoms and degenerative changes at presentation, and for the otherwise inexplicable failures that the patient presented.

**Fig. 8:**
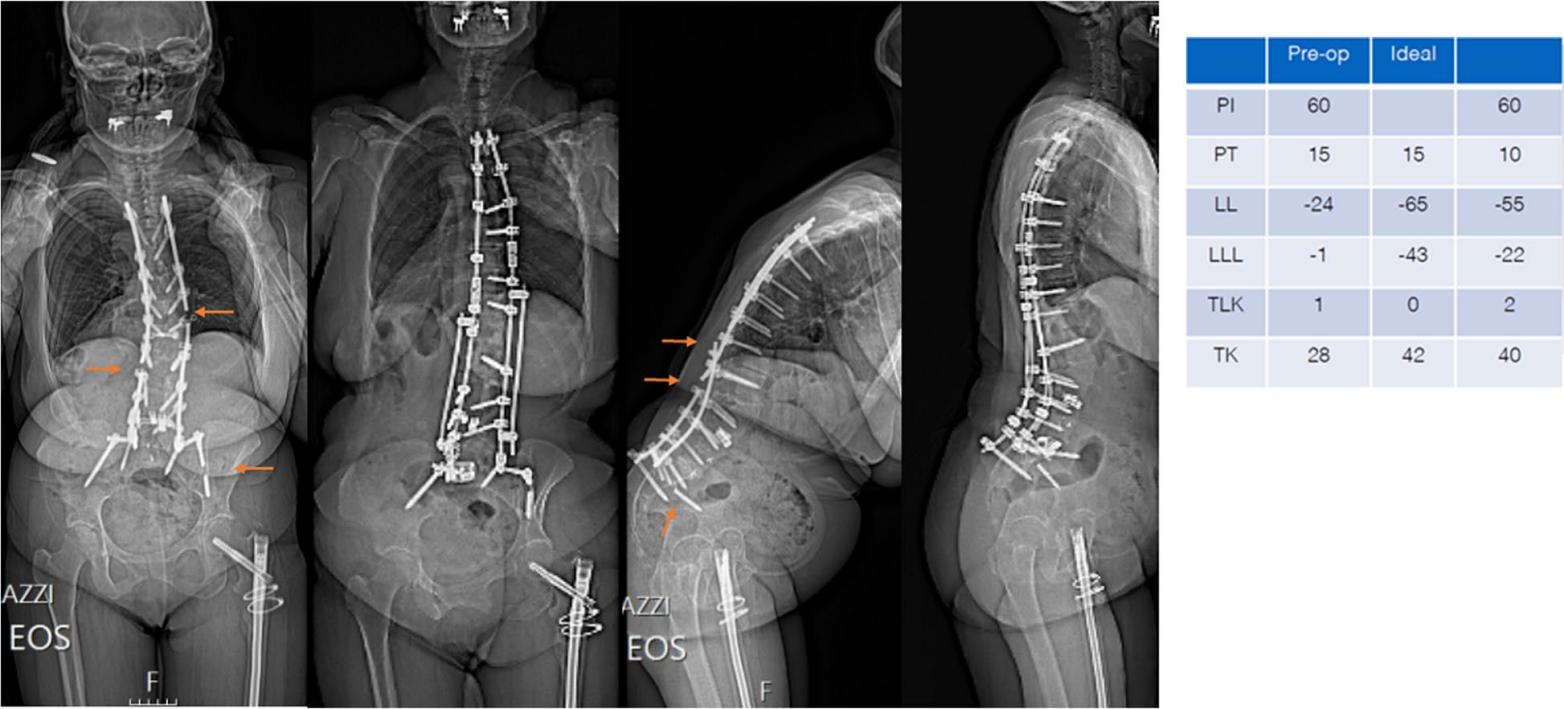
Pre- and postoperative x-ray following revision surgery in January 2021. The preoperative imaging showed multiple rod breakages and the breakage of the left iliac screw (arrows). A T4-pelvis revision with implant renewal, posterior release, hyperlordotic ALIF and 4-rod-construct was performed. After revision surgery LL and TK were restored. However, a deficit in LLL was observed

The x-rays performed in October 2019 showed multiple rod breakages along with the breakage of the left iliac screw (Fig. 8): revision surgery was planned but it was delayed due to the Covid 19 pandemic. The operation was conducted in January 2021 and once again the sagittal balance could be restored (Fig. 8). At the last surgery, a 4-rod construct was employed with an ALIF cage to restore the sagittal balance and maximize the stability of the implant. At the last follow up the obtained sagittal balance was maintained and there was no sign of subsidence of the ALIF cage.

A timeline of the evolution of the clinical course is provided in Table 1.

**Table 1:** Overview of the timeline of the clinical history with summary of the symptoms, most relevant imaging and treatment

Date	Symptoms	Imaging	Treatment
June 2002	Low back pain	X-rays: early-stage degenerative scoliotic curve	Conservative Treatment
September 2003	Increased low back pain and left sciatic pain	X-rays and CT scan: marked progression of the deformity with endplate erosion	Conservative Treatment
April 2007	Increased symptoms and functional impairment	X-rays: further progression of the deformity	L1-L4 posterior fusion with L3/4 PLIF
December 2012	Back pain and sagittal imbalance	X-rays: distal junction kyphosis	T8-pelvis posterior fusion with L2 pedicle subtraction osteotomy and TLIF L4/5 and L5/S1
March 2013	Increasing axial pain and coronal and sagittal imbalance	CT scan: subsidence of both TLIF cages, T8 fracture	T7-pelvis posterior fusion
October 2019	Increasing axial pain and sagittal imbalance	X-rays: multiple rod breakages and breakage of the left iliac screw	Revision with 4-rod construct and ALIF cage L5/S1

## Discussion and conclusions

The presented case report illustrates the difficulties encountered in the diagnosis and treatment of spinal RDOA, offering possible strategies to overcome them. While this pathologic condition was first described for the hip joint already in 1959 [5], very little research has been done around the topic, and even less so in spine setting, with the present case being the first one of spinal RDOA reported in the literature. While eight cases of “destructive discovertebral disc disease” have been observed in a previously published paper, the lack of rapid destruction and the presence of comorbidities such as advanced osteoporosis, rheumatoid arthritis or breast cancer in this patient cohort suggest that this case represents a different pathologic entity from the ones already reported [6].

**Fig. 9:**
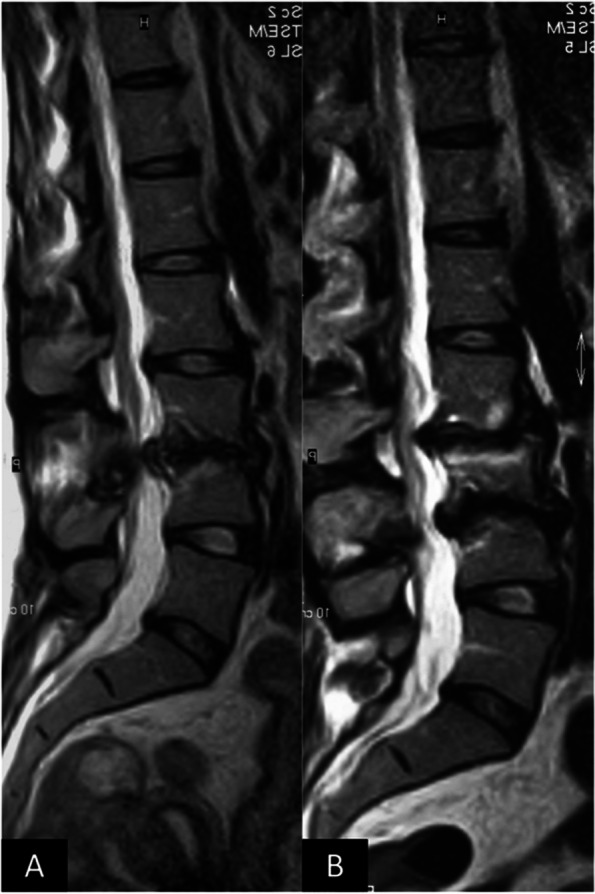
**A **and** B**: comparison of T2-weighted MRI scans from September 2003 (**A**) and April 2007 (**B**). Figure B shows destruction and osteophyte formation of the L3/4 disk space, the first one involved in the pathologic process, and bone edema in L2/3, which appeared healthy in the previous imaging (Figure A)

Similarly to what has been described for atraumatic hip RDOA, the presented patient is an adult female, although younger than the observed age range for hip RDOA (47–90 years) [1]. The diagnosis of RDOA for hip and shoulder is based on the radiographic imaging. However, the concepts known for these clinical settings were easily recognizable in the spine as well. The first stage of RDOA is characterized by bone marrow edema in T2 MRI images with possible subchondral fractures [7–11]. Within months from the first diagnosis, the disease evolves to bone destruction and remodeling with extensive osteophyte formation [1], or disk space in the spine. At this stage, bone destruction is evident in the x-rays as well. This evolution is visible in the MRI imaging conducted in April 2007 for the presented patient (Fig. 9): the L3/4 disk, the first one being involved in the pathologic process as visible in Fig. 2 (CT scan from September 2003), shows extensive destruction and osteophyte formation, while the L2/3 disk, which was not initially involved, presented bony edema of the endplates. Figure 9 shows clearly how spinal RDOA is an evolving process, that progressively involves adjacent disk spaces. This observation is confirmed also by the subsidence of the TLIF cages in L4/5 and L5/S1 in the x-rays from March 2013 (Fig. 7), which was probably caused by the destruction of the endplates due to RDOA progression. The timeline of the evolution of the disease is highlighted in Table 2. The observation that RDOA is an evolving disease has obvious relevance for the planning of the therapy and for the informed consent of the patient. It is fundamental to make the patient aware of the fact that disease progression to adjacent segments may cause an evolving spine deformity and may possibly require multiple surgeries over time.

**Table 2:** Schematic representation of the evolution of spine RDOA and its charachteristic radiographic findings

Stage	Radiographic findings
1	T2-weighted MRI images show bone marrow edema and possibly subchondral fractures of the endplates
2	Within months, X-rays show extensive remodelling of the disc space with bone destruction and osteophyte formation
3	Involvement of adjacent disk spaces

While the exact pathogenesis of RDOA is still being investigated, a recent study evidenced a higher concentration of serum bone turnover markers and a higher osteoclastic activity in patients affected by hip RDOA [12]. In fact, the SPECT scan performed in 2003 for the presented patient to rule out a malignancy showed a higher signal at L3/4, the first involved level. The bone mineral density in hip RDOA was similar to that of patients with “regular” osteoarthritis or femoral neck osteonecrosis [13]. Serum tartrate-resistant acid phosphatase 5b (TRACP-5b) and bone alkaline phosphatase (BAP) have been proposed as possible markers of hip RDOA [12, 13] and may potentially aid the diagnosis of spinal RDOA as well. However, further studies are required to investigate this point.

As we have observed in this case, the correct and timely diagnosis of spinal RDOA is key for a targeted surgical and therapeutical planning and for the informed consent of the patients. Thanks to the correct postoperative sagittal alignment, multiple implant failures and relapses of the sagittal imbalance would have not been expected in a patient with an unimpaired bone structure and bone metabolism. This was the observation that led us to look for a further explanation for the clinical course of this patient, as the recurrent complications could not be explained by regular osteochondrosis or adjacent segment degeneration alone. The exclusion of an infectious, rheumatologic, metabolic or malignant disease leaves spinal RDOA as the only viable diagnosis. This diagnosis is confirmed by the available imaging, which is coherent with the characteristics of hip and shoulder RDOA. While a single case is not sufficient to determine precise diagnostic criteria, we suggest that any cases of rapid evolving destruction of the disk space should be treated as spinal RDOA. A bone edema around the endplates in T2 MRI images followed by destruction of the disk space and osteophyte formation in less than a year’s time is, in our opinion, strongly suggestive of this condition. In the future we hope that specific serum markers will be developed to confirm the diagnosis of RDOA.

Due to the progression of the pathology even following fusion surgery, not only at the adjacent segments but at the index level as well, the choice of type of interbody fusion is of particular relevance in spinal RDOA. The use of smaller cages that are not supported by the ring apophysis, such as those used for PLIF or TLIF, might provide a less than ideal load distribution and stability in the setting of spinal RDOA. In the presented case, both PLIF and TLIF cages subsided despite a correct sagittal alignment, probably because of the impaired structure and metabolism of the cancellous bone. While it is not known to what extent the ring apophyses are affected by RDOA, the use of larger cages such as ALIF or LLIF might be a safer and more reliable option in this setting [14–17]. The dramatic mechanical complications observed in this case suggest that spinal RDOA cases may pose more stringent requirements to implant stability and durability than standard degenerative cases. Satisfactory correction of misalignment with proximal and distal ends of the implant in neutral load zones (close to the gravity line), multiple fixation points and multiple rod constructs may help reduce risk of revision surgery [18]. Multi-rod constructs have shown to reduce the rate of pseudoarthrosis and mechanical complications in adult deformity surgery [19, 20]. When the surgical therapy of spinal RDOA requires long fusion, the use of multi-rod constructs may improve the posterior load sharing thus limiting the risk of cage subsidence. The follow-up available in this case supports these observations.

If the hypothesis of a hyperactivation of the osteoclasts in RDOA was confirmed, surgical therapy may be supported by pharmacological management with osteoclasts inhibitors or inhibitors of the local bone renin-angiotensin system to reduce the risk of disease progression and cage subsidence [21, 22]. However, this option is purely hypothetical and is not supported by scientific evidence yet.

The main limitation of this work is the presentation of a single case. However, as this clinical setting seems very uncommon, the difficulties experienced in the treatment of this patient will help clinician identify spinal RDOA and consider the peculiarities of the bone structure impairment to plan surgery accordingly.

To conclude, spinal RDOA is a newly defined clinical entity that is characterized by a quickly developing edema of the vertebral endplates followed by a destruction of the disk space and osteophyte formation within months from the first diagnosis. These changes are clearly shown in MRI imaging and, later, in x-rays. The diagnosis is based solely on the radiographic findings. This disease is not associated to other causes of rapidly progressive degeneration such as malignancy, rheumatic disease, osteonecrosis or infection. Spinal RDOA progresses in the involved segment and to the adjacent disks despite surgical therapy. The surgical treatment should take the impaired bone structure into accountcon and biomechanically sound constructs including appropriate selection of proximal and distal instrumented vertebrae, large interbody cages and multi-rod constructs should be taken into consideration when planning the operation. In the future, pharmacological therapy may support the surgical management and limit the progression of the disease.

## Data Availability

not applicable.

## Abbreviations

ALIF: Anterior lumbar interbody fusion
BAP: Bone alkaline phosphatase
DJK: Distal junction kyphosis
LLIF: Lateral lumbar interbody fusion
PLIF: Posterior lumbar interbody fusion
RDOA: Rapidly destructive osteoarthritis
RPOA: Rapidly progressive osteoarthritis
TLIF: Transforaminal lumbar interbody fusion
TRACP-5b: Erum tartrate-resistant acid phosphatase 5b
